# Mining massive genomic data of two Swiss Braunvieh cattle populations reveals six novel candidate variants that impair reproductive success

**DOI:** 10.1186/s12711-021-00686-3

**Published:** 2021-12-16

**Authors:** Irene M. Häfliger, Franz R. Seefried, Mirjam Spengeler, Cord Drögemüller

**Affiliations:** 1 Institute of Genetics, Vetsuisse Faculty, University of Bern, Bremgartenstrasse 109a, 3001 Bern, Switzerland; 2 Qualitas AG, 6300 Zug, Switzerland

## Abstract

**Background:**

This study was carried out on the two Braunvieh populations reared in Switzerland, the dairy Brown Swiss (BS) and the dual-purpose Original Braunvieh (OB). We performed a genome-wide analysis of array data of trios (sire, dam, and offspring) from the routine genomic selection to identify candidate regions showing missing homozygosity and phenotypic associations with five fertility, ten birth, and nine growth-related traits. In addition, genome-wide single SNP regression studies based on 114,890 single nucleotide polymorphisms (SNPs) for each of the two populations were performed. Furthermore, whole-genome sequencing data of 430 cattle including 70 putative haplotype carriers were mined to identify potential candidate variants that were validated by genotyping the current population using a custom array.

**Results:**

Using a trio-based approach, we identified 38 haplotype regions for BS and five for OB that segregated at low to moderate frequencies. For the BS population, we confirmed two known haplotypes, BH1 and BH2. Twenty-four variants that potentially explained the missing homozygosity and associated traits were detected, in addition to the previously reported *TUBD1*:p.His210Arg variant associated with BH2. For example, for BS we identified a stop-gain variant (p.Arg57*) in the *MRPL55* gene in the haplotype region on chromosome 7. This region is associated with the ‘interval between first and last insemination’ trait in our data, and the *MRPL55* gene is known to be associated with early pregnancy loss in mice. In addition, we discuss candidate missense variants in the *CPT1C*, *MARS2*, and *ACSL5* genes for haplotypes mapped in BS. In OB, we highlight a haplotype region on chromosome 19, which is potentially caused by a frameshift variant (p.Lys828fs) in the *LIG3* gene, which is reported to be associated with early embryonic lethality in mice. Furthermore, we propose another potential causal missense variant in the *TUBGCP5* gene for a haplotype mapped in OB.

**Conclusions:**

We describe, for the first time, several haplotype regions that segregate at low to moderate frequencies and provide evidence of causality by trait associations in the two populations of Swiss Braunvieh. We propose a list of six protein-changing variants as potentially causing missing homozygosity. These variants need to be functionally validated and incorporated in the breeding program.

**Supplementary Information:**

The online version contains supplementary material available at 10.1186/s12711-021-00686-3.

## Background

Good female fertility in cattle is of high interest in agriculture, to maintain production and genetic diversity within a population [1]. Therefore, traits that affect growth, birth, and fertility are important to breeders and semen suppliers [1]. In the current Swiss breeding programs, the traits used or intended for breeding value estimation are e.g., non-return rate, the time between calving, birth weight, calving ease, multiple birthing, rearing success, traits regarding survival within a defined period, and slaughter weight. Several studies have shown that fertility ability in dairy cattle has declined in international breeds [2, 3], the main reason being the unfavourable correlation between milk production and fertility [2–4]. This has led to increased efforts to include and prioritize fertility traits in breeding schemes and to identify the genetic causes in female reproduction, in order to breed more fertile cows. Generally, these traits have a low heritability, however, with the improvement of genomic methods, including the implementation of genomic selection (GS) [5], the reliability of breeding values can be increased [6]. Genomic selection is based on single nucleotide polymorphism (SNP) array genotyping and is especially interesting for highly complex traits with a low to moderate heritability, since it can partially account for Mendelian sampling [5, 6]. Combining comprehensive genotyping of breeding animals and recording of the phenotypes within a breeding program provides a promising database for genetic evaluations, including genome-wide association studies (GWAS). These can be carried out for all recorded phenotypes included in a breeding scheme to identify the underlying quantitative trait loci (QTL). Furthermore, the genetic variation of the population is well represented within the routinely genotyped cattle population. Studies on the search for haplotypes that show a significant reduction in homozygosity have been carried out in many species and breeds (e.g. [7–27]). Haplotypes that show no homozygous carriers may indicate the presence of recessive lethal variants within these regions. In this way, many causative variants have been detected by mining these haplotypes and using whole-genome sequencing data (e.g. [9–18, 26–32]). In the international Brown Swiss population, two haplotype regions have been repeatedly mapped, namely Brown Swiss Haplotype 1 (BH1) on chromosome 7 and Brown Swiss Haplotype 2 (BH2) on chromosome 19 [11, 19]. For BH2, which is associated with high juvenile mortality, a likely causative variant was found in the *TUBD1* gene [11, 19]. Likewise, various embryonic lethal protein-changing variants have been reported to be associated with haplotypes showing missing homozygosity in other cattle populations [10–18, 27, 33]. These examples demonstrate that short-read-based whole-genome sequencing (WGS) data make it possible not only to identify genomic regions, but also to decipher the entire genetic code of an individual in a time- and cost-efficient manner. Thereby the likely causative variants for monogenic traits segregating in livestock populations can be discovered and systematically selected against [23].

In Switzerland, Original Braunvieh (OB) and Brown Swiss (BS) are agronomically important dual purpose and dairy cattle populations, respectively. Historically, the OB population is the autochthonous brown Swiss cow with no BS influence and is adapted to the Swiss climate, whereas the modern BS originates from the OB by introgression of North American BS cattle [34]. It has been shown that the two populations segregate genetically from each other and are thus considered independent populations [35]. Both populations occur predominantly in the eastern parts of Switzerland and still have an important role in traditional alpine farming, which is frequently practiced.

The aims of this study were to identify haplotypes associated with growth, birth and fertility traits in two local Braunvieh cattle populations. Based on missing homozygosity screens that focus on trios, linkage disequilibrium analyses, and trait associations, we detected both known and novel haplotype regions segregating at low to moderate frequencies with a negative influence on female reproduction and calf survival. Based on WGS data, we identified potential protein-coding candidate causative variants for selection, including those that impair early embryonic and pre-weaning survival.

## Methods

**Fig. 1:**
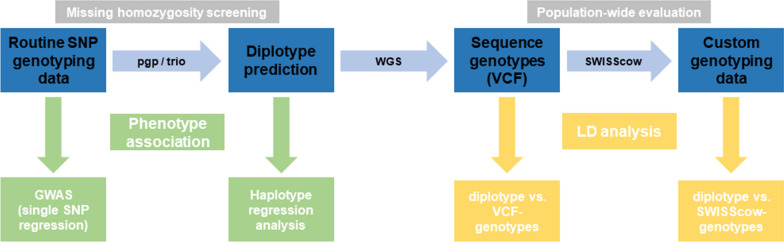
Workflow of the reverse genetic analyses. Note that the steps of the main genomic analyses that are described in the Methods section are indicated in blue. Further evaluations are shown in green (phenotypic analysis) and yellow (linkage disequilibrium (LD) analysis)

To give a comprehensive overview of the methods applied, the workflow used to mine the genomic data from SNP arrays, WGS data, and a broad variety of phenotypes for the Swiss BS and OB populations is summarized in Fig. 1.

### SNP array data

The SNP array data used in this study were provided by the breeding associations of the BS and OB populations. The cleaned SNP dataset comprised 114,890 SNPs and represents a combination of different SNP arrays. As of 2018, the positions of SNPs were updated to the latest cattle reference sequence ARS-UCD1.2 [36, 37]. Data were imputed by using the software Fimpute v2.2 [38] to correct for erroneously called SNPs and to complete the SNP set for individuals genotyped with a lower chip density. Data were phased by using the Beagle v5.0 software package [39].

The OB dataset included 10,085 genotyped animals, from which 3287 trios (trio: sire, dam, and offspring) and 4360 paternal half-sib groups (pgp: **p**arent – **g**rand-**p**arent; sire, maternal grandsire, and offspring) were derived for the analyses (Table 1). The BS dataset included 48,807 genotyped animals, with 14,450 trios and 32,319 half-sib groups (Table 1). The SNP array data were quality filtered by retaining SNPs with a MAF higher than 0.01 and a call rate greater than 0.9, and individuals with a call rate greater than 0.8.

**Table 1:** Number of genotyped animals from the two Braunvieh cattle populations used for missing homozygosity scan, haplotype, and GWAS analyses

	Original Braunvieh (OB)	Brown Swiss (BS)
Number routine genotyped animals	10,085	48,807
Number trios (sire, dam and offspring)	3287	14,450
Number paternal half sib groups—pgp (sire, maternal grandsire and offspring)	4360	32,319

### Haplotype analysis

We used the snp1101 software to screen the genomic data for haplotypes that showed a significant deviation from the Hardy–Weinberg equilibrium (HWE) based on the exact test of HWE [40, 41]. The screen was performed for each population and repeated for both the trio-based and the pgp-based approaches. The dataset used for screening was generated by including the genotypes of the Swiss animals born after 2009 and their ancestors. The snp1101 software screens each autosome based on a sliding-window approach, for which we defined a window size of 50 SNPs that represents genome regions ranging from 0.26 to 3.2 Mb with an average length of 1.1 Mb. The output is a list of all the significant haplotypes with the number of observed and expected homozygous carriers, the allele frequency and the haplotypes themselves. We carefully defined all the regions of interest as the regions that show a reduction in homozygosity and a significant deviation from HWE with their adjusted p-value for a false discovery rate (FDR) corrected according to the Benjamini-Yekutieli procedure [42]. For each region, we chose the most significant haplotype to reduce the number of false positive carriers. We defined the diplotype status for each haplotype of all the animals in the population to select the most relevant animals for whole-genome sequencing. By sequencing healthy breeding animals that are either heterozygous or homozygous carriers of the diplotypes, we increased the probability of sequencing animals that carry the associated causal variants.

### Association studies

Based on the predicted diplotype status, haplotype association studies were carried out for a variety of traits and for each of the observed haplotypes. For the association analyses, routinely available conventional, non-genomic breeding values related to fertility, birth, and growth were used. The individual traits are described in Table 2. Only breeding values with a reliability higher than 0.5 were deregressed according to Garrick et al. [43] and used in a linear mixed model using the genome-wide complex trait analysis (GCTA) software [44]. Thereby, only the animals that have own performance or progeny performances were included in the analysis. The model is as follows: ${y}_{EBV}=\mu +G+\mathbf{H}{\varvec{\upbeta}}+\epsilon$, where ${y}_{EBV}$ is the deregressed breeding value (drEBV), $\mu$ is the average of the deregressed breeding values, $G$ is a random genetic effect based on a genomic relationship matrix (GRM) estimated by the GCTA software [44], $\mathbf{H}$ is a vector of the diplotype status per animal, ${\varvec{\upbeta}}$ is a vector of the estimated fixed effects of each haplotype and $\epsilon$ accounts for random variation.

**Table 2:** The 24 female fertility traits analysed

Trait group	Trait sub-group	Trait	Description
Fertility traits	Fertility	Non-return rate heifer (nrr)	Heifers non-return rate after 56 days, binary
		Non-return rate cow (nrk)	Cows non-return rate after 56 days, binary
		Interval first to last insemination heifer (vzr)	Interval between first and last insemination for heifer, days
		Interval first to last insemination cow (vzk)	Interval between first and last insemination for cows, days
		Interval calving to insemination (raz)	Interval from calving to first service, days
Birth traits	Birth history direct	Percentage normal births (ngd)	Calving ease, scored between 1-without help to 5-dystocia
		Percentage live births (lgd)	Percentage of calves born alive
		Birth weight (ggd)	Weight of calve at birth, kg
		Gestation length (tdd)	Days from successful insemination to birth
		Multiple birth (twind)	Percentage of multiple births
	Birth history maternal	Percentage normal births (ngm)	Calving ease, scored between 1-without help to 5-dystocia
		Percentage live births (lgm)	Percentage of calves born alive
		Birth weight (ggm)	Weight of calve at birth, kg
		Gestation length (tdm)	Days from successful insemination to birth
		Multiple birth (twinm)	Percentage of multiple births
Growth-related traits	Rearing success	Survival period 1 (p1)	Survival from day 3 up to day 30
		Survival heifer period 2 (hp2)	Survival of heifers from day 31 up to 458 days
		Survival bull period 2 (bp2)	Survival of young bulls from 31 days up to 183 days
	Slaughter traits calves	Slaughter weight (cwco)	Weight at slaughter, kg
		Carcass conformation score (ccco)	Amount of meat at slaughter, kg
		Carcass fat score (cfco)	Fat cover in the meat
	Slaughter traits adults	Slaughter weight (cwao)	Weight at slaughter, kg
		Carcass conformation score (ccao)	Amount of meat at slaughter, kg
		Carcass fat score (cfao)	Fat cover in the meat

We also performed genome-wide association studies (GWAS) on the same genomic and phenotypic data used above and we calculated the association of each single SNP to the deregressed breeding value using the model: ${y}_{EBV}=\mu +G+S{\varvec{\upbeta}}+\epsilon$ , where ${y}_{EBV}$ is the drEBV, $\mu$ is the average of the drEBV, $G$ is a random effect based on a GRM estimated by the method in [45], $S$ is a scalar of the genotype status of each SNP per animal, ${\varvec{\upbeta}}$ is the estimated fixed effect of each SNP and $\epsilon$ accounts for random variation. For this analysis, we used the snp1101 software [40] and applied a Bonferroni correction for multiple testing with an increased significance level at p < 4.35e−7. All the genotyped animals were included in these models. The GWAS results were visualized by creating Manhattan plots with the qqman package [46].

### Preparation of the whole-genome sequencing data

To identify the putative causative variants, we performed whole-genome sequencing on selected animals that were predicted to be carriers of the identified haplotypes. For this purpose, we sequenced 70 animals in addition to the 360 genomes that had been sequenced within other projects or which are publicly available, to reach a total of 430 genomes of healthy breeding animals (see Additional file 1: Table S1). Among these, 114 and 100 were from purebred BS and OB animals, respectively. First, the reads were trimmed with fastp [47] by applying the following quality thresholds with the flags: --qualified_quality_phred 20, --length_required 35, --cut_window_size 3, --cut_mean_quality 15, --cut_by_quality5 20, --cut_by_quality3 20, and for the WGS data from the NovaSeq6000, the flag --trim_poly_g was added. The quality-controlled reads were mapped to the latest cattle reference sequence ARS-UCD1.2 [36, 37] using the Burrows-Wheeler Aligner v. 0.7.17 [48], deduplicated by using the MarkDuplicates tool from the Picard software v.2.18.2 [49], recalibrated using the BaseRecalibrator and PrintReads applications of the GATK v3.8.1.0.gf15c1c3ef software [50] with known variants from the 1000 Bull Genomes Project run 7 (BQSR file version 2) [51], and sorted by using the samtools v1.8 software [52].

The average read depth and insert size were calculated using covstats in the goleft v0.1.19 tool [53]; read depth ranged from 6.8 × to 72.8×, with an average of 18.6x. For genotyping, the HaplotypeCalling, CombineGVCFs, CatVariants and GenotypeGVCFs tools from GATK v3.8.1.0.gf15c1c3ef were used to produce a variant call format (VCF) file [50]. To predict the effects of the detected variants, we used the NCBI Annotation Release 106 [54] and the SnpEff v4.3 software [55]. Furthermore, quality scores were estimated for each variant by applying the GATK recommendation for hard-filtering of indels and small nucleotide variants (SNV) with the SelectVariants and VariantFiltration tools of GATK v3.8.1.0.gf15c1c3ef [50]. This workflow is consistent with the workflow proposed by the 1000 Bull Genomes Project (run 7) [51, 56].

### Analysis of the WGS data for the design of the custom array

An algorithm was implemented to efficiently screen the identified haplotype regions with a significant depletion in homozygous animals for candidate variants. Thus, we increased the window size by 2 million bp on each side and we looked for variants that did not occur in the homozygous state in any of the 430 animals. As the read depth varied, we allowed for missing values (a maximum of 50% of genotypes) but assumed that all variants passed the quality score criteria. In addition, we postulated that at least one animal in each population, BS or OB, had to be a carrier of potential variants, but that not more than 75% of the animals should be carriers.

Subsequently, we designed a custom Affymetrix array called "SWISScow chip" in cooperation with the Swiss breeding associations. We selected 465,768 variants to design this custom array, of which 236,043 were selected from the haplotype regions mapped in this study and 230,305 were protein-coding variants spread genome-wide. The design process allowed us to consider 44% of the initially selected variants. The final SNP panel encompassed 318,216 variants, including 112,854 "routine" variants that were genotyped before using different Illumina bovine beadchips plus 205,362 project-specific variants, so called "research" markers.

This array was used in the Swiss routine genotyping throughout 2020, and provided us with the genomic information of 13,667 animals including 6575 BS and 1489 OB plus a cohort of 5603 Swiss Holstein (HO) cattle.

### Linkage disequilibrium (LD) analyses

To confirm the association between the variants in the VCF file and the defined haplotypes, we calculated the linkage disequilibrium between the defined haplotypes’ diplotypes and the variants in the VCF file, and with the genotypes from the custom array. While linkage analysis of the VCF file included 79 BS and 94 OB samples, further analysis depended on the quality control performed on the VCF data of the chromosome of interest (plink function --mind 0.1) [57]. This information was integrated into the data from the custom array, including the 8064 Braunvieh genotypes. Linkage analyses were performed using the plink v1.9 software [57].

### Visualization of the results

**Fig. 2:**
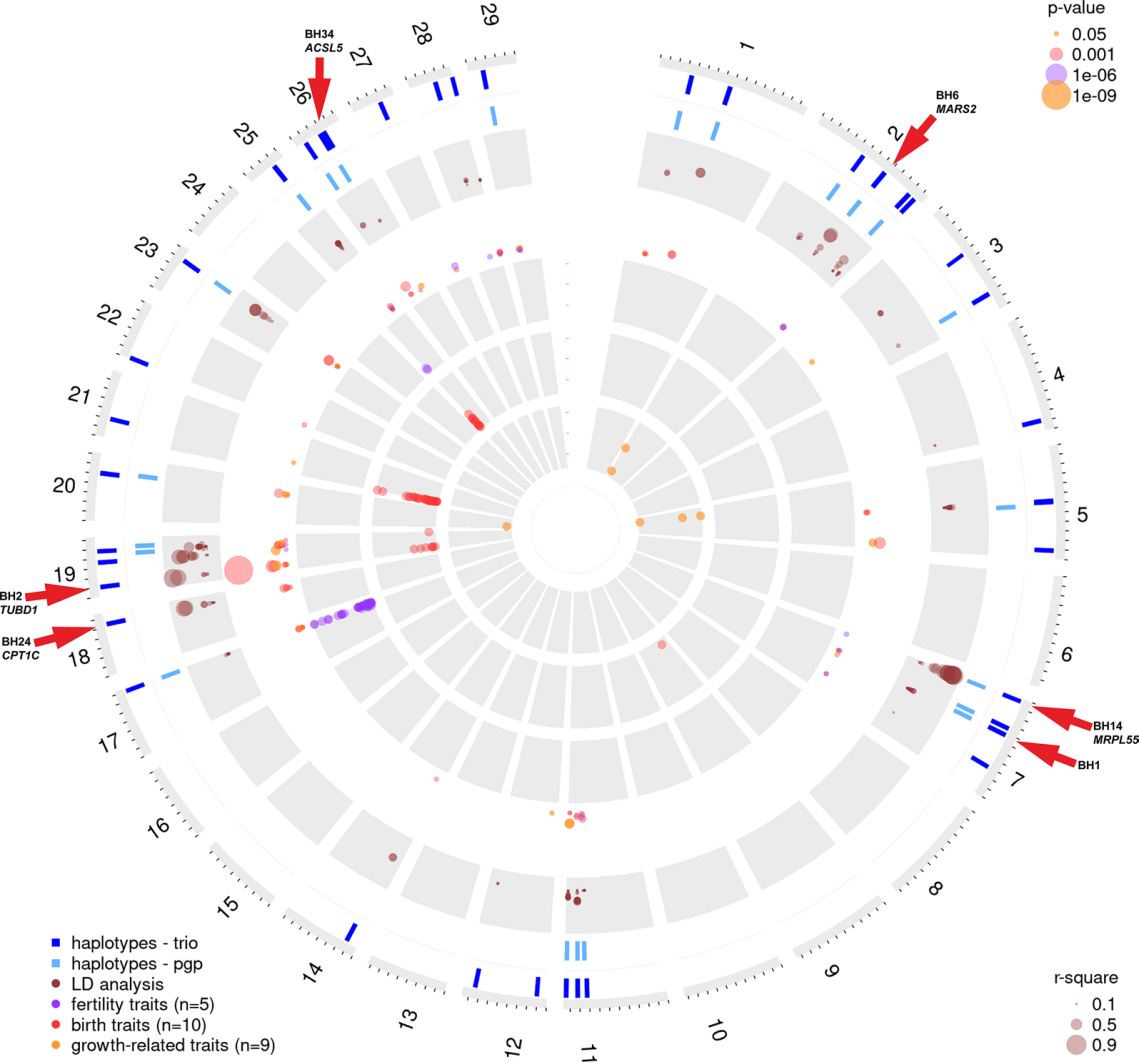
Genome-wide summary of the data mining for the Brown Swiss population. In the outer circles, the identified haplotypes per chromosome with reduced homozygosity for the trio-based approach are indicated in dark blue and their associated haplotypes of the pgp-based approach in light blue. Note that only the haplotypes that were detected through the pgp-based approach are shown if, within the same region, another haplotype was detected by the trio-based approach. The circle with the brown dots indicates LD (r^2^) between haplotypes and markers on the custom SNP array. Note that the dot size correlates with the extent of LD. The third circle shows the significant haplotype association results. Note that the different colors represent the three groups of evaluated traits and the dot size correlates with the significance values accordingly. The three inner circles present the significant GWAS results across all fertility (purple), birth (red), and growth-related (yellow) traits. Scales are based on the −log10(p-value). Note that the red arrows indicate the previously identified haplotypes BH1 and BH2 [19] and the herein described *MRPL55*-related haplotype BH14, as well as the BH14, BH24 and BH34 haplotypes and their associated genes that harbor the most likely causative variants

**Fig. 3:**
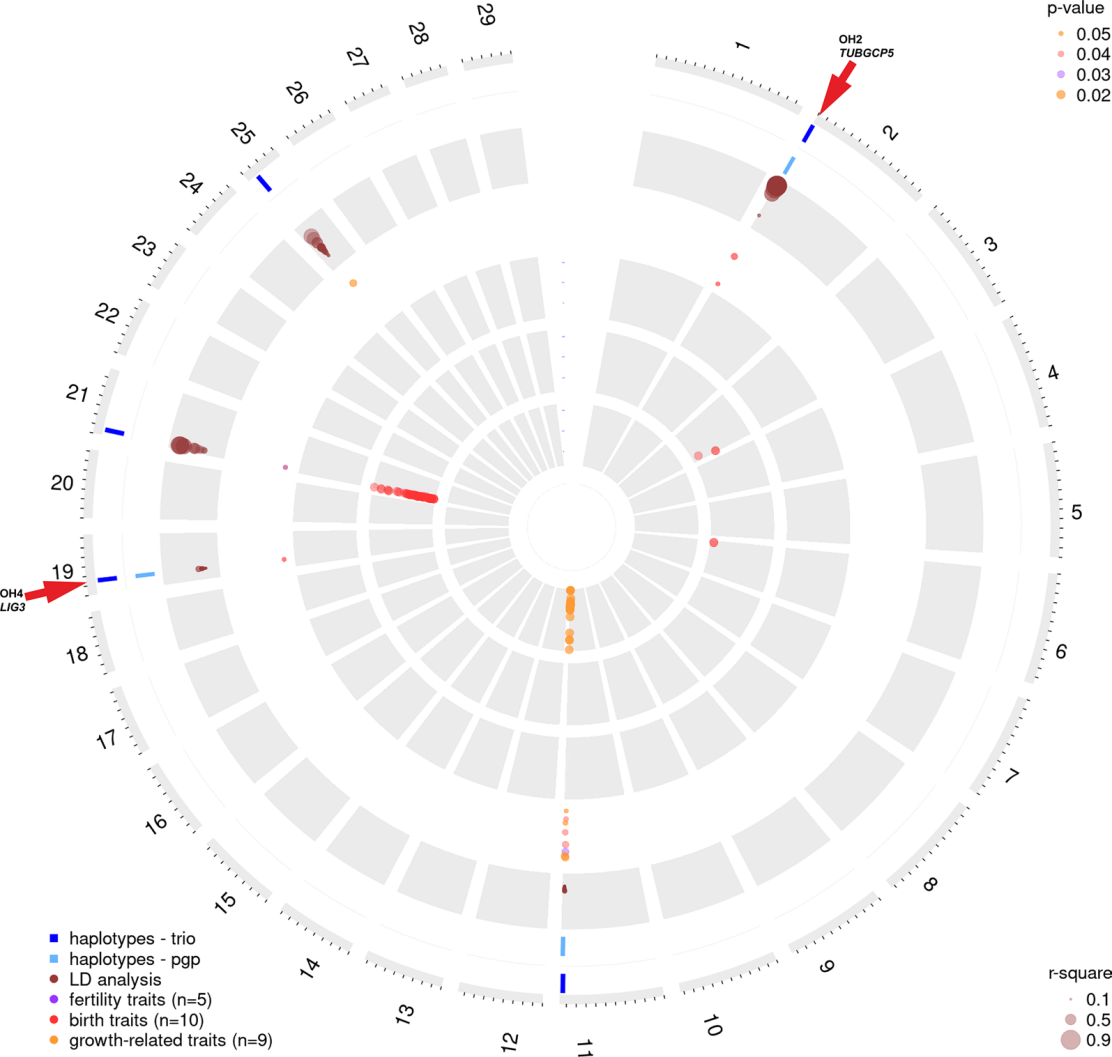
Genome-wide summary of the data mining for the Original Braunvieh population. In the outer circles, the identified haplotypes per chromosome with reduced homozygosity for the trio-based approach are indicated in dark blue and their associated haplotypes of the pgp-based approach in light blue. Note that only the haplotypes that were detected through the pgp-based approach are shown if, within the same region, another haplotype was detected by the trio-based approach. The circle with the brown dots indicates LD (r^2^) between haplotypes and markers on the custom SNP array. Note that the dot size correlates with the extent of LD. The third circle shows the significant haplotype association results. Note that the different colors represent the three groups of evaluated traits and the dot size correlates with the significance values accordingly. The three inner circles present the significant GWAS results across all fertility (purple), birth (red), and growth-related (yellow) traits. Scales are based on the −log10(p-value). Note that the red arrows indicate the *LIG3*-related haplotype OH4 described in this paper, as well as the OH2 haplotype and its associated gene *TUBGCP5*

To provide a comprehensive overview of the results, the OmicCircos Rpackage was used to visualize the identified genomic regions [58]. Figures 2 and 3 were obtained by including the genomic regions of all significant haplotypes from the trio approach and, if in the same haplotype region, a significant haplotype with the pgp approach was found, this is indicated in the plots. Furthermore, the plots include the LD results between markers on the custom array with the diplotypes, the significant haplotype association for the various traits, and the significant GWAS association combined in the above-defined trait groups (Table 2) for BS and OB, respectively.

### Interpretation of the variants

For the interpretation of the genomic variants, the UCSC Genome Browser tool suite was used to calculate conservation scores [59]. First, the LiftOver UCSC tool and their LiftOver file for BosTau9 to HG38, which is the DNA base conversion from the bovine reference sequence ARS-UCD1.2 [36] to the human genome 38 [60], was applied by command-line coordinate, lifting all the variants from the VCF file. Furthermore, for these new positions, the base-wise conservation scores of 99 vertebrate genomes with the human genome were extracted from the UCSC database and resulted in the phyloP and PhastCons scores shown in Table 3 and Table S5 (see Additional file 6: Table S5) [61, 62]. Both scores represent a comparative genomic alignment approach, but they use different algorithms. PhyloP measures the conservation and evolutionary acceleration at individual sites with absolute values of −log(p-value), and PhastCons identifies stretches of conservation with values demonstrating probabilities of negative selection from 0 to 1 [61, 62].

**Table 3:** List of haplotypes in the Brown Swiss population identified by the trio-based approach

Name	Chr	Start	End	Allele frequency	Proposed associated gene^a^
BH1^b^	7	41371808	42545291	3.02	*TCF3*
BH2^c^	19	9726237	10819756	4.22	*TUBD1*
BH3	1	34908448	36034631	2.05	
BH4	1	76587512	77861430	2.20	*GMNC*
BH5	2	57787148	58768161	2.87	*LRP1B*
BH6	2	86065338	87460373	3.41	*MARS2*
BH7	2	120284002	121473182	1.93	*SPATA3*
BH8	2	127933873	129004898	2.98	
BH9	3	52432936	52,689742	1.43	*HFM1*
BH10	3	101636810	102827915	2.26	
BH11	4	108811998	109953666	2.66	
BH12	5	57653479	59777422	2.32	*RNF41* and *LRP1*
BH13	5	110617654	111664596	1.63	
BH14	7	2588873	3357718	3.04	*MRPL55*
BH15	7	33954389	34956540	4.12	
BH16	7	79845977	80708046	2.20	
BH17	11	82034654	82881173	2.39	*PGGHG*
BH18	11	91766016	92804942	2.54	
BH19	11	103891372	105003404	1.77	
BH20	12	10254014	10967287	2.02	
BH21	12	74954939	75817277	1.61	
BH22	14	12290759	13350202	2.64	
BH23	17	69786512	70645226	2.98	
BH24	18	52977041	54113281	2.32	*CPT1C*
BH25	19	35808547	36844178	1.86	*SNF8*
BH26	19	47874708	48724082	1.89	
BH27	20	50058036	51370414	2.56	
BH28	21	19368258	20362870	1.60	
BH29	22	311724	1277064	2.07	
BH30	23	39600366	40609442	2.49	
BH31	25	27713801	29132187	1.80	*ITGAD* and *SPN*
BH32	26	9215188	10180467	1.72	
BH33	26	26659976	27689471	2.72	
BH34	26	31353340	32429589	2.37	*ACSL5*
BH35	27	28524504	29550764	1.80	
BH36	28	26661955	27743768	1.75	
BH37	28	45215132	45913154	2.07	
BH38	29	14497752	15554643	3.08	

*BH* BS haplotype, *Chr* chromosome number, *Start and End* start and end positions according to the reference sequence ARS-UCD1.2 [36]

^a^According to NCBI Annotation Release 106 [54]

^b^Previously described haplotype [19]

^e^Previously described haplotype and associated gene [11, 19]

For the variants that show a linkage between the WGS data and the haplotypes in relevant regions, we extracted information about their effects by manually using the web-based prediction tools PROVEAN [63] and MutPred2 [64] resulting in the SiftScores and MutPred2 scores shown in Table S5 (see Additional file 6: Table S5).

Finally, we analysed the segregation of candidate variants within the international cattle population of the 1000 Bull Genomes project run 8 that includes 4109 animals from various breeds [56].

## Results

### Detection of numerous novel haplotypes associated with fertility, birth, and growth-related traits

We identified many genomic candidate regions that show a significant depletion in homozygosity, significant haplotype association, and a genome-wide association to fertility, birth, and growth-related traits. For the two Braunvieh populations studied here, BS and OB, each of the haplotype analyses based on the pgp- and the trio-based approaches resulted in a concatenated list of all the haplotypes that deviated significantly from HWE. The identified haplotypes were named according to previous studies [19] as BH haplotypes for BS and OH haplotypes for OB (Tables 3 and 4). For the BS population, we detected 38 haplotypes with the trio-based approach (Table 3) and 53 haplotypes with the pgp-based approach, with 19 overlapping haplotypes (Fig. 2) and (see Additional file 2: Table S2). Two of the identified haplotypes, BH1 on chromosome 7 and BH2 on chromosome 19 had already been detected in 2011 [19] (Fig. 2). The haplotypes that deviated most significantly from HWE were BH6, BH14, and BH1, with no homozygous carriers although at least 44 were expected (see Additional file 2: Table S2). For the OB population, we detected five significant novel haplotypes with the trio-based approach (OH2 to OH6, see Table 4) and three haplotypes with the pgp-based approach, which all overlapped with those detected by the trio-based approach (Fig. 3) and (see Additional file 2: Table S2). The haplotypes that deviated most significantly from HWE were OH2 and OH4, with no observed homozygous diplotype carriers, although at least 15 were expected (see Additional file 2: Table S2). After correction for FDR, three interesting haplotype regions (OH7, OH8 and OH9) with a non-significant depletion of homozygosity were found for OB (see Additional file 2: Table S2; Additional file 3: Table S3; and Additional file 5: Table S4).

**Table 4:** List of haplotypes in the Original Braunvieh population identified by the trio-based approach

Name^a^	Chr	Start	End	Allele frequency	Proposed associated gene^b^
OH2	2	1005580	1614673	4.48	*TUBGCP5*
OH3	11	10,406494	104418358	5.76	*MYMK*
OH4	19	14336760	15222429	3.87	*LIG3*
OH5	21	5195518	6367707	4.56	*LYSMD4*
OH6	25	9596610	10624288	3.87	*USP7*

*OH* OB haplotype, *Chr* chromosome number, *Start and End* start and end positions according to the reference sequence ARS-UCD1.2 [36]

^a^Since the name OH1 is reserved for a pathogenic *CNGB3*-variant [101], numbering starts at OH2

^b^According to NCBI Annotation Release 106 [54]

The complete GWAS results for the 24 traits, conducted on the identified BH and OH haplotypes are in Table S3 (see Additional file 3: Table S3). BH and OH haplotypes showed significant associations on 21 and five different chromosomes, respectively (Figs. 2 and 3). The strongest significant association was found for BH2 with four birth and two growth-related traits (see Additional file 3: Table S3), which confirms the previous finding that the BH2-related missense variant p.His210Arg in the *TUBD1* gene causes ill-thrift and juvenile mortality in Brown Swiss cattle [11].

All the results of the single SNP GWAS are visualized in traditional Manhattan plots (see Additional file 4: Fig. S1) and the most significant markers were selected for a comprehensive overview per trait group (Figs. 2 and 3) and (see Additional file 5: Table S4). For example, obvious GWAS hits for fertility traits in BS, such as non-return rate and interval first to last insemination are on chromosome 17 where BH23 was detected, and very strong GWAS signals for birth traits in OB, such as calving ease, birth weight, and gestation length, are on chromosome 21, where OH5 was found. Among the studied growth-related traits, we identified a highly significant association signal on chromosome 11 in OB, which affects carcass conformation and co-localizes with the OH3 haplotype.

### Potential candidate causative variants in *MRPL55* and* LIG3*

Subsequently, we mined the genome-wide sequence data to identify potential causative candidate variants responsible for either prenatal lethality or postnatal lethality, or sub-lethal phenotypes. Analysis of the variants that show depletion in homozygosity and are in LD with the haplotype identified protein-changing variants that are potentially causative in five BH and two OH haplotypes (Tables 5 and 6). Genotyping based on the SWISScow custom array allowed to validate whether three of the BS variants were present in several thousands of young animals. As for the haplotypes, we detected animals with homozygous variant genotypes in the current population, which lead us to anticipate either that late-onset undesired traits might occur or that these variants have incomplete penetrant effects. The disorders known to be associated with the affected genes range from embryonic lethality (e.g., *MRPL55* [65] and *LIG3* [66]) and metabolic diseases (e.g. *acyl-CoA synthetase long chain family member 5* (*ACSL5)* [67]) to neurodevelopmental disorders (e.g., *methionyl-tRNA synthetase 2* (*MARS2*) [68]) (Tables 5 and 6).

**Table 5:** Short list of five potential candidate causative variants for the Brown Swiss population^a^

Haplotype-region information		Gene	OMIM/OMIA	Associated disorder/gene function	Variant designation^b^			
Name	Approach				Genomic position	Transcript	Coding DNA change	Protein change
BH2	Trio	*TUBD1*^c^	607344/001939-9913	Juvenile mortality	chr19:10833921	NM_001075470.2	c.629A>G	p.His210Arg
BH6	Trio and pgp	*MARS2*	609728	Spastic ataxia 3 (lethal)	chr2:86191230	NM_001098971.1	c.1553G>A	p.Arg518Gln
BH14	Trio and pgp	*MRPL55*	611859	Early pregnancy loss	chr7:2996436	NM_001303490.1	c.169C>T	p.Arg57*
BH24	Trio	*CPT1C*	608846	Spastic paraplegia (lethal)	chr18:56098048	XM_002695120.5	c.158G>A	p.Gly53Asp
BH34	Trio	*ACSL5*	605677/002226-9615	Lipid malabsorption	chr26:32940521	NM_001075650.1	c.528C>G	p.Asn176Lys

*Chr* chromosome, *BH* BS haplotype

^a^Comprehensive list available in Table S5 (see Additional file 6: Table S5)

^b^According to the reference sequence ARS-UCD1.2 [36] and NCBI Annotation Release 106 [54]

^c^Previously described variant [11]

**Table 6:** Short list of two potential candidate causative variants for the Original Braunvieh population^a^

Haplotype-region information		Gene	OMIM/OMIA	Associated disorder/gene function	Variant designation^b^			
Name	Approach				Genomic position	Transcript	Coding DNA change	Protein change
OH2	Trio and pgp	*TUBGCP5*	608147	Proper formation of the mitotic spindles	chr2:1268426	NM_001102495.1	c.311C>A	p.Thr104Lys
OH4	Trio and pgp	*LIG3*	600940	Embryonic lethality	chr19:15080335	NM_001038107.2	c.2483_2484 + 4delAGGTGC	p.Lys828fs

*Chr* chromosome, *OH* OB haplotype

^a^Comprehensive list available in Table S5 (see Additional file 6: Table S5)

^b^According to the reference sequence ARS-UCD1.2 [36] and NCBI Annotation Release 106 [54]

We specifically selected one variant on chromosome 7 at ~ 3 Mb in the BS population that is predicted to be deleterious due to a stop-gain variant in the *MRPL55* gene. This mutation introduces a premature stop codon (p.Arg57*), which truncates the encoded protein by almost 80% (Table 5). This variant is in perfect LD (r^2^ = 1) with the BH14 haplotype in the WGS animals and in high LD (r^2^ = 0.9) in the custom array genotyped animals. It was never observed in the homozygous state in any of the BS individuals. Furthermore, BH14 shows a significant negative association with the fertility trait ‘interval first to last insemination’ in cows and a mentionable but non-significant negative association with the fertility trait ‘interval calving to first insemination’ and the growth-related trait ‘survival of young bulls’ (see Additional file 3: Table S3). Evaluation of the *MRPL55*-related variant with the custom array revealed that it segregates within the BS population at a frequency of 0.032 without any homozygous carriers and deviates significantly from HWE (see Additional file 6: Table S5). In the OB population with more than 1400 animals genotyped with the SWISScow custom array, genotyping found only two heterozygous animals for this variant. In addition, none of the 4109 animals from various breeds within the run 8 data of the 1000 Bull Genomes project were homozygous for this variant [51].

In the BS population, we identified three additional missense variants in the *carnitine palmitoyltransferase 1C* (*CPT1C*), *MARS2*, and *ACSL5* genes (Table 5), which affect highly conserved nucleotides. Their effects on protein level were predicted to be deleterious by PROVEAN and pathogenic by MutPred2 (see Additional file 6: Table S5). Unfortunately, only one of these variants was included in the SWISScow custom array, which was associated with BH24 and located in the *CPT1C* gene (p.Gly53Asp). This variant was never detected in the homozygous state in the WGS data, and only a few homozygous carrier animals were detected in the SWISScow genotypes (see Additional file 6: Table S5). The BH24 haplotype is associated with several birth traits such as birth weight, percentage of normal births, and percentage of live births. The p.Arg518Gln variant in the *MARS2* gene that is associated with the BH6 haplotype is also of interest since this haplotype was never detected in the homozygous state. Unfortunately, this variant was not considered in subsequent array-based genotyping. Lastly, the p.Asn176Lys variant in the *ACSL5* gene that is associated with the BH34 haplotype was also never detected in the homozygous state within the available WGS data and, to date, has not been genotyped in other animals. BH34 was rarely observed in the homozygous state in the BS population and is statistically negatively associated with the growth-related traits ‘heifer survival after 30 days’ and ‘carcass fat score in calves’.

In the OB population, we draw attention to the haplotype region OH4 located on chromosome 19 at ~15 Mb. For this haplotype, we identified a frameshift variant (p.Lys828fs) in the *LIG3* gene leading to the change of 90 residues and premature termination of translation compared to the wild-type protein sequence, which is 26 residues longer (Table 6). This variant is in perfect LD (r^2^ = 1) with the OH4 haplotype in the WGS animals. OH4 is negatively associated with the percentage of live births. Unfortunately, this frameshift variant was not part of the SWISScow custom array. Nevertheless, the *LIG3* variant segregates at an allele frequency of 0.035 in the WGS data of Braunvieh animals. In addition, none of the 4109 animals from various breeds within the run 8 data of the 1000 Bull Genomes project [51] were homozygous for this variant. According to OMIM #600940, the *LIG3* gene is essential for DNA repair of mitochondrial DNA.

A missense variant that affects an evolutionary highly conserved residue (p.Thr104Lys) in the *tubulin gamma complex associated protein 5* (*TUBGCP5*) gene was detected in the OB population but was never observed in the homozygous state in the WGS data (see Additional file 6: Table S5). The associated OH2 haplotype has a negative effect on multiple births and a positive effect on the percentage of normal births. Although absent from the SWISScow array, this variant segregates at an allele frequency of 0.021 in the WGS data and is in perfect LD with the haplotype (see Additional file 6: Table S5).

In addition, for 13 other BH and two other OH haplotypes, we propose candidate causative variants (see Additional file 6: Table S5), but most of them are predicted to have a neutral or benign effect or represent synonymous variants.

## Discussion

This comprehensive study explored the genomic data of the two current Swiss Braunvieh dairy populations BS and OB, for reduced homozygosity due to hidden recessive variants. Such variants which, for the most part, change the amino acid sequence of proteins, lead to natural or artificial selection against homozygous individuals. This phenomenon could be due to embryonic lethality, reduced rearing success, or exclusion from the breeding population due to poor development. To better understand the functional role of the associated haplotype regions, we performed genome-wide missing homozygosity scans and subsequent comprehensive association analyses.

In the recent past, various studies that aimed at pinpointing such haplotype regions used routine SNP genotyping data and the standard approach (pgp) in which the paternal grandfather replaces the usually non-genotyped dam [19, 21, 27]. Here, we chose a trio-based approach, assuming that it would increase the power of the statistical analysis of haplotypes due to the direct relationships of the families analyzed [21]. The trio-based approach resembles the transmission ratio distortion approach used to search for lethal alleles, however, the latter is based on Bayesian statistics, while we applied the Fisher exact test of HWE [7, 8, 24, 41]. Especially for the OB population for which the amount of available data is limited, the trio-based approach is notably more efficient than the standard analysis, since more regions with lower haplotype frequencies were detected. In contrast, for the BS population, the pgp-based approach, which considers more than twice the number of family groups, detected more regions than the trio-based analysis. However, it should be kept in mind that an increased number of detected haplotype regions might include more false positives, which is supported by our results with some of the haplotype regions detected in the BS population not showing any phenotypic associations. Therefore, we focused on the haplotype regions that were detected by the trio-based approach and showed phenotypic associations with the 24 analyzed fertility, birth, and growth-related traits.

To validate the chosen approach, we searched for previously identified haplotypes and known causal recessive variants and confirmed the BH1 and BH2 haplotypes in the studied BS population [11, 19]. BH2 is known to be negatively associated with fertility and growth-related traits. We also confirmed that the variant in the *TUBD1* gene showed the highest level of LD with BH2. Interestingly, although breeding against this variant has been practiced for several years, we were still able to detect this haplotype region and its effects on the studied traits. However, strong selection of the bulls has successfully eliminated the two inherited disorders spinal muscular atrophy [69, 70] and weaver syndrome [14, 31, 71] since we did not detect any carriers. Regarding the OB population, it is known that the Fleckvieh haplotype 2 (FH2) and the associated frameshift variant in the *SLC2A2* gene segregate in the OB population at an allele frequency of 0.05 and cause the Fanconi-Bickel syndrome with growth retardation [9, 72]. Nonetheless, we did not detect the region surrounding this variant on chromosome 1. The reason why we could not find a significant depletion in homozygosity for FH2 was that the genotyping data included several homozygous carriers of the variant associated with the Fanconi-Bickel syndrome. Due to the fact that this disorder is non-lethal, but induces liver and kidney defects leading to reduced growth [9], apparently normally developed new-born calves are genotyped before the manifestation of growth retardation.

Our aim was to detect novel causative variants, but first we confirmed the detection of the BH1 haplotype region previously reported in BS cattle [19, 33]. We found evidence for depletion of homozygotes, but a positive association with the growth-related trait carcass fat score and no indication of a negative effect on fertility traits as previously described [33]. We suggest that it is caused by the p.Asp70Asn missense variant that affects a highly conserved nucleotide in the *transcription factor 3* (*TCF3)* gene, also known as *E2A*. In men, mutations in this gene are associated with agammaglobulinemia 8 (OMIM #616941) (see Additional file 6: Table S5). *Tcf3* knock-out mice show growth retardation and increased neonatal death [73]. TCF3 is associated with the Wnt signaling pathway and thereby influences osteogenesis and is essential for B cell differentiation [73, 74]. This same variant was previously reported to be associated with BH1, but with inconclusive results [33]. In our study, the estimated LD between BH1 and this variant is rather low and the haplotype associations are merely suggestive. Therefore, either this variant causes retarded growth and increased pre-weaning lethality, but with incomplete penetrance, or is irrelevant as not all loss-of-function variants have a pathogenic effect [75]. We found several dozens of homozygous carriers of the *TCF3* variant, although it segregates with an allele frequency of 0.11 and deviates significantly from HWE. Therefore, we recommend to keep track of this variant, e.g. by clinically examining live homozygous individuals.

Regarding our extensive list of newly identified haplotype regions in the Swiss Braunvieh populations studied here, we propose six candidate causative variants (Tables 5 and 6), which are all supported by high LD values with the corresponding haplotype, and 17 variants of interest (see Additional file 6: Table S5). In the future, for many other mapped haplotypes, the detection of such variants should be done with the same approach based on population-wide evaluation, because unfortunately only every second variant is technically designable for the custom array. Furthermore, it is disputable if instead of array-based genotyping with its known limitations, one could consider genotyping by sequencing by using low-pass sequencing [76, 77]. Nonetheless, this recent method has also strong limitations such as a restricted probability to see the variant of interest on the sequence of a given individual as well as the need to be followed by imputation that is not accurate for recent variants due to the existence of ancestral and derived versions of identical haplotypes. Due to the large evidence gained by the analysis of massive phenotypic and genomic data, we recommend further evaluation of such haplotype regions and candidate variants in the future.

Among the variants suggested to cause reduced omozygosity in the BS population, we highlighted the *MRPL55* nonsense variant on bovine chromosome 7 associated with BH14. Statistically, this variant fits perfectly with the expectations for embryonic lethal variants, since LD is very high and there are no homozygous animals. The *MRPL55* gene belongs to the mitochondrial ribosomal protein (MRP) gene family, more precisely to the MRPL group, which are genes that make up the large subunit of the mitoribosome complex [78]. The mitoribosome complex is a multi-gene complex of diverse genes, denoted MRP genes [65]. Mouse knock-out experiments have revealed that different genes of the MRP family cause early embryonic death at the stage of pre-gastrulation, and expression analyses of MRP genes have shown their essential role during embryogenesis [65, 79]. Furthermore, mRpL55 was found to have a crucial role during the development of fruit flies, with mRpL55 null individuals showing increased mortality, reduced growth and activity after hatching [80]. Due to the nature of the bovine variant, two-thirds of the truncated protein are lacking, with most likely non-sense mediated decay taking place, thus it can be assumed that the variant leads to a loss-of-function of *MRPL55* in the homozygous state. Finally, this could explain putative early embryonic lethality and is consistent with the complete lack of homozygous animals. Moreover, the described biological effect is supported by the association study indicating negative associations with fertility traits and a reduction in heifer survival. The latter can be explained with fertility issues being one of the major culling reasons in dairy cattle [81, 82].

For the BS population, we propose three additional variants as candidate causal variants impairing postnatal survival. First, the BH24-associated missense variant in the *CPT1C* gene, which is known to be important for the proper development of the brain [83], was predicted to have a deleterious effect on protein level and effects on an evolutionary highly conserved nucleotide. Disorders associated with pathogenic *CPT1C* variants cause lethal spastic paraplegia (OMIM #616282). The BH24 haplotype shows significant associations with several birth traits and therefore this variant represents a plausible candidate for reduced homozygosity, which was confirmed after targeted genotyping. Second, the BH6-associated most likely pathogenic missense variant in *MARS2*, a gene that plays an important role in embryonic development, is analogous to *MRPL55,* potentially leading to developmental arrest during early embryogenesis [79]. In humans, variants in this gene are associated with mitochondrial respiratory chain disorders and lead to retarded growth and hypotonia [84], and are also associated with a neurodevelopmental disorder (OMIM #611390) [68]. Third, the most likely pathogenic BH34-associated missense variant in *ACSL5*, which is involved in lipid metabolism, might cause a developmental delay due to disturbed fat metabolism in various mammalian species (OMIM #605677, OMIA #002226-9615) [67], and might explain the negative effects that we observed on growth-related traits. Both the *MARS2* and *ACSL5* variants need to be evaluated by further genotyping to confirm the postulated effects.

For the OB population, we propose two novel variants as candidate causal variants impairing embryonic survival. First, the OH4-associated frameshift variant in the *LIG3* gene, which is related to early embryonic lethality in mice [66], is of high interest. Functionally, LIG3 is a DNA binding ligase that is responsible for the repair of strand breaks [85, 86]. However, more precisely, the function of LIG3 is not essential for the repair of nuclear DNA since other proteins can compensate for the lack of LIG3, but it is essential for the repair of mitochondrial DNA [85, 86]. Second, the OH2-associated missense variant in the *TUBGCP5* gene, although not predicted as having a damaging effect on an evolutionary highly conserved nucleotide, it might represent a suitable candidate, since *TUBGCP5* plays an important role in the γ-tubulin ring complex which binds to the centrosome and thereby affects the cell cycle [87–89]. This complex has a highly conserved structure including six γ-tubulin proteins. TUBGCP5, in contrast to the other proteins, is present in single copy in the γ-tubulin ring complex [87–89]. In humans, an association between a *TUBGCP5* missense variant and microcephaly, which is a severe anomaly, has been shown [90]. Although to date these two variants have not been evaluated in the broader population, the observed significant negative effect of the OH2 haplotype on the maternal birth trait ‘percentage of live births’ and the deleterious nature of the *LIG3* and *TUBGCP5* variants support the assumed negative impact on reproduction.

Furthermore, three additional haplotypes (OH7, OH8, and OH9) which showed no significant HWE-deviation after correcting for FDR were detected. Interestingly, we found very convincing candidate variants for these haplotypes, such as the missense variant in the *HSD3B7* gene located on bovine chromosome 25. This variant is in high LD with OH9 and no homozygous carrier animals were found. *HSD3B7* is associated with the often lethal recessively inherited bile acid synthesis defect (BASD; OMIM #607764) and with progressive liver disease characterized by malabsorption of lipids and vitamins [91, 92]. *HSD3B7* knock-out mice showed increased mortality of homozygous newborns and decreased cholesterol absorption in the surviving animals [92]. Given the nature of the bovine *HSD3B7* missense variant and the observed negative effect on the growth-related trait ‘bull survival’, we speculate that homozygous mutant animals are most likely either non-viable or impaired in development.

Interestingly a unique haplotype region on bovine chromosome 11 was mapped in both populations, suggesting a common causative variant and therefore underlining the common heritage of the two Swiss Braunvieh cattle populations [34, 35]. Nevertheless, previous studies in OB have identified this region as a signature of selection [35, 93]. For these haplotypes (BH19 and OH3), we have not identified any potentially causative variant. The most probable variant identified was a missense variant in the *myomaker (MYMK)* gene; however, this variant occurs in the homozygous state in many animals. Therefore, it is likely a less suitable candidate for the depletion of homozygosity. Interestingly the OB-specific GWAS results for the growth-related carcass conformation score traits pinpoint the same region on chromosome 11 at 104 Mb. Based on the function of the *MYMK* gene on skeletal muscle development [94], we suggest that the impact of this variant on economically important growth traits needs to be subsequently evaluated. In contrast, deletion of *Mymk* in mice is perinatal lethal [94]. This example illustrates nicely the case of a variant that potentially underlies balancing selection as shown previously in pigs and cattle [95, 96]. Alternatively, and more likely, other undetected variants, such as more complex structural variants, might be responsible for the observed depletion in homozygosity in this genomic region.

As described above, not all the variants that we claim to be potentially causing disease could be evaluated in the current population, as many of them were unfortunately not included on the custom array. There are technical reasons why many variants were not included on this final array, such as interference with adjacent variants, high GC-content, repetitive DNA, multi-allelic variation, etc. Nonetheless, the SWISScow custom chip proved to be a very powerful and efficient method to validate the segregation of the most likely causative variants in the current population. Furthermore, the genomic data of a population changes over time since the haplotype frequencies can change due to the impact of popular sires. Regarding the haplotype regions, we may have failed to detect some specific haplotype regions because the window size was set at 50 markers, since Hoff et al. [25] showed that the homozygosity rate negatively correlates with window length.

Although we propose 24 new variants, including six of high importance, the causal variants for numerous haplotype regions including BH1 remain unclear. For example, in the BS population, we identified the highly HWE-deviating haplotype BH23 on chromosome 17, which showed significant haplotype associations with growth-related and birth traits, plus suggestive associations with fertility. In parallel, for the same region, suggestive GWAS associations with the fertility trait ‘non-return rate’ were observed, but we failed to identify candidate causal variants.

This analysis is based on the latest reference sequence ARS-UCD1.2 of a Hereford cow, however, the reference sequence still spans many gaps and includes more than 2000 unplaced scaffolds that could potentially harbor important coding sequences [37]. Recently, it was shown that reference graphs based on sequence data of several breeds can include new functional sequences [97]. Another drawback of our approach is that we restricted our analysis to coding variants, while non-coding variants impairing the expression of a protein or more complex structural variants disturbing the function of genes might also be causative. The interference of non-detected structural variants could also be an explanation for the rather low LD values in some regions [98, 99]. Furthermore, as a few homozygous diplotype carriers are alive, some haplotypes may arise due to the high selective pressure on traits within the breeding program. As shown by the example of cholesterol deficiency in Holstein cattle, the associated haplotype can segregate in the population under two indistinguishable versions, a derived variant and its ancestral version [100]. In summary, the reported haplotype regions might reflect population-specific characteristics of the breeding programs, such as the intensive use of individual bulls by artificial insemination or by natural service, a common practice in Swiss OB. Most likely, the focus applied on monogenic Mendelian disorders is too simplistic since a polygenic architecture can often be assumed. The latter is supported by the GWAS results that highlight numerous additional associated genomic regions, which are not affected by a depletion of homozygosity.

## Conclusions

For the first time, we applied the trio-based mapping approach in cattle for the genome-wide detection of haplotypes showing reduced homozygosity that segregate at low to moderate frequencies. We present a short list of potentially causative variants and highlight four coding variants for the Brown Swiss population and two for the Original Braunvieh population located in candidate genes that are involved in embryonic and pre-weaning lethality. Although it will be challenging to evaluate all the candidate variants that we propose here by targeted monitoring of at risk matings and possible clinical examination of live homozygotes, it is important to rule out causality. This study illustrates the difficulty to select for improved fertility and rearing success by focusing on monogenic disorders, since our GWAS results confirmed the polygenic nature of these traits. Nonetheless, the proposed candidate causative variants will help to refine DNA-based selection decisions to improve female fertility and rearing success in Swiss Braunvieh cattle.

## Supplementary Information

**Additional file 1: Table S1.** WGS sample information. All whole-genome sequencing samples included in this study and their project and sample ids from the European Nucleotide Archive (ENA). In addition, the sex, breed and average read depth and insert size are provided.

**Additional file 2: Table S2.** Haplotype information. All haplotypes for the trio- and pgp-based approaches for the BS (**A**) and the OB population (**B**). The descriptive data include the type of analysis, genomic positions, number of expected and observed animals, haplotype frequency, p-value from exact tests of Hardy–Weinberg equilibrium and the adjusted p-value according to the Benjamini-Yekutieli procedure.

**Additional file 3: Table S3. **Output of the haplotype analyses. Results of the estimation of the haplotype effect with different conformation, production and reproduction traits for the BS (**A**) and OB populations (**B**).

**Additional file 4: Figure S1. **Manhattan plots and their QQ-plots of the GWAS results for the BS and OB populations. There is a page for each of the fertility, birth and growth-related trait groups, including a Manhattan plot for every single trait according to Table 2.

**Additional file 5: Table S4.** GWAS results. Significant (p < 4.35e−7) GWAS results for the BS (**A**) and (**B**) OB populations.

**Additional file 6: Table S5.** Comprehensive list of candidate causal variants. The data for BS (**A**) and OB (**B**) are provided, with information regarding haplotypes and the potential candidate causal variants based on the reference sequence ARS-UCD1.2 [36] and the NCBI Annotation Release 106 [54], including variant effect predictions (siftScore [63] and MutPred2 score [64]) and base conservation scores (phyloP and phastCons [61, 62]). Furthermore, the genotype distributions across whole-genome sequencing and genotyping data are included.

## Data Availability

For access to the genotyping data, interested people are asked to contact the Braunvieh breeding association directly.

## Abbreviations

*ACSL5*: *Acyl-CoA synthetase long chain family member 5*
Arg: Arginine
BASD: bile acid synthesis defect
BH: Brown Swiss Haplotype
BH1: Brown Swiss Haplotype 1
BH2: Brown Swiss Haplotype 2
bp: base pairs
BS: Brown Swiss
*CPT1C*: *Carnitine palmitoyltransferase 1C*
Cys: Cysteine
drEBV: deregressed estimated breeding value
FDR: False discover rate
FH2: Fleckvieh Haplotype 2
GS: genomic selection
GWAS: genome-wide association study
HD: high density SNP panel
HO: Holstein
*HSD3B7*: *Hydroxy-delta-5-steroid dehydrogenase, 3 beta- and steroid delta-isomerase 7*
HWE: Hardy–Weinberg equilibrium
LD: low density SNP panel
*LIG3*: *DNA ligase 3*
*MARS2*: *Methionyl-tRNA synthetase 2, mitochondrial*
Mb: Mega bases
*MRPL55*: *Mitochondrial ribosomal protein L55*
*MYMK*: *Myomaker*
OB: dual-purpose breed Original Braunvieh
OH: Original Braunvieh Haplotype
pgp: parent – grant parent (paternal half-sib groups)
QTL: quantitative trait loci
r2: R-squared value of the LD analysis
*SCFD2*: *Sec1 family domain containing 2*
*SLC2A2*: *Solute carrier family 2 member 2*
SNV: small nucleotide variants
SNP: single nucleotide polymorphism
SNP array: genotyping arrays including single nucleotide polymorphisms
*TCF3*: *Transcription factor 3*
TRD: transmission ratio distortion
*TUBD1*: *Tubulin delta 1*
*TUBGCP5*: *Tubulin gamma complex associated protein 5*
VCF: variant call format file
WGS: whole-genome sequencing
