# Supplementary material for: Mining massive genomic data of two Swiss Braunvieh cattle populations reveals six novel candidate variants that impair reproductive success
Source: Genet Sel Evol. 2021 Dec 16;53:95. doi: 10.1186/s12711-021-00686-3 (PMC8675516; doi:10.1186/s12711-021-00686-3)

Additional file 4: **Figure S1.** Manhattan plots and their QQ-plots of the GWAS in BS and OB. There is a page for the fertility, birth and growth-related trait group, including a Manhattan plot for every single trait in the trait group according to Table 2.

BS fertility traits

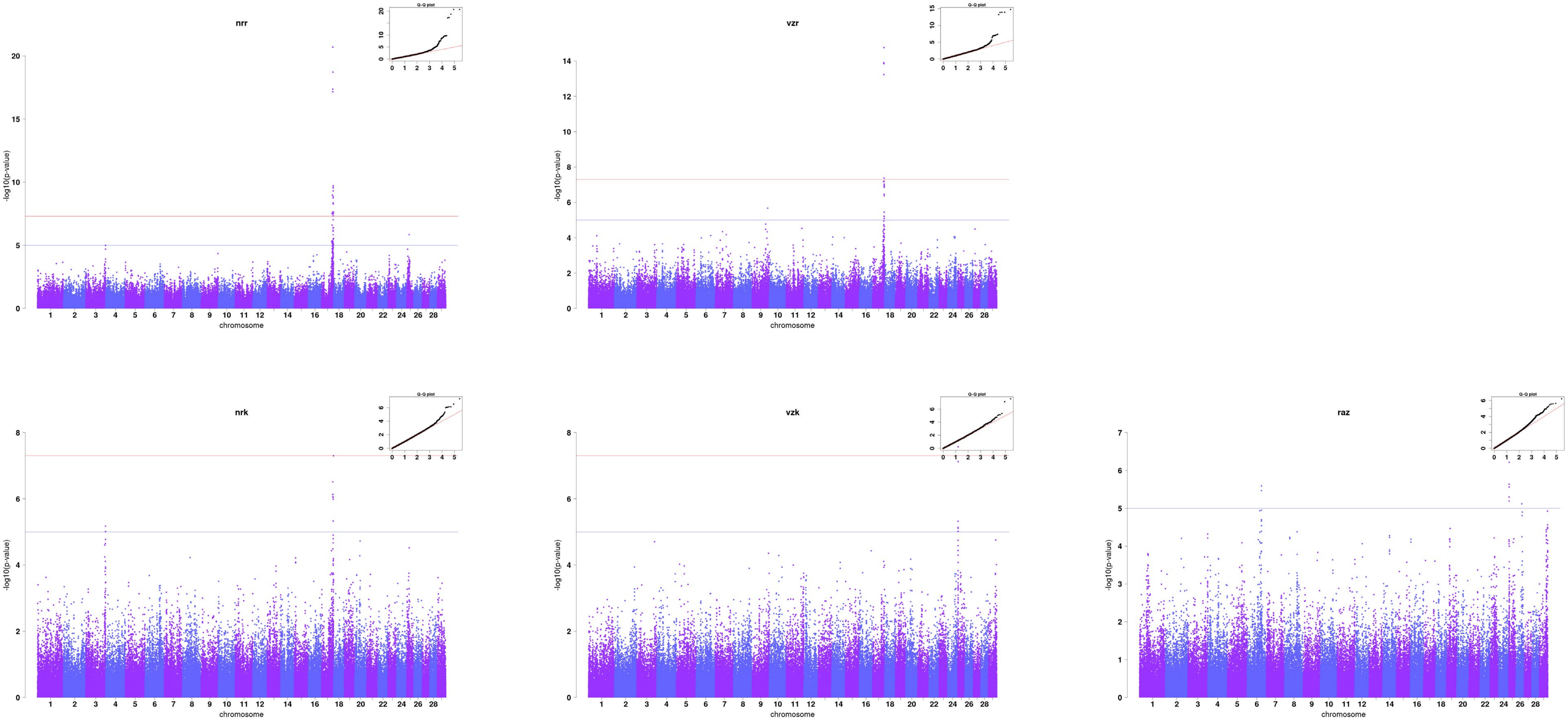

BS birth traits

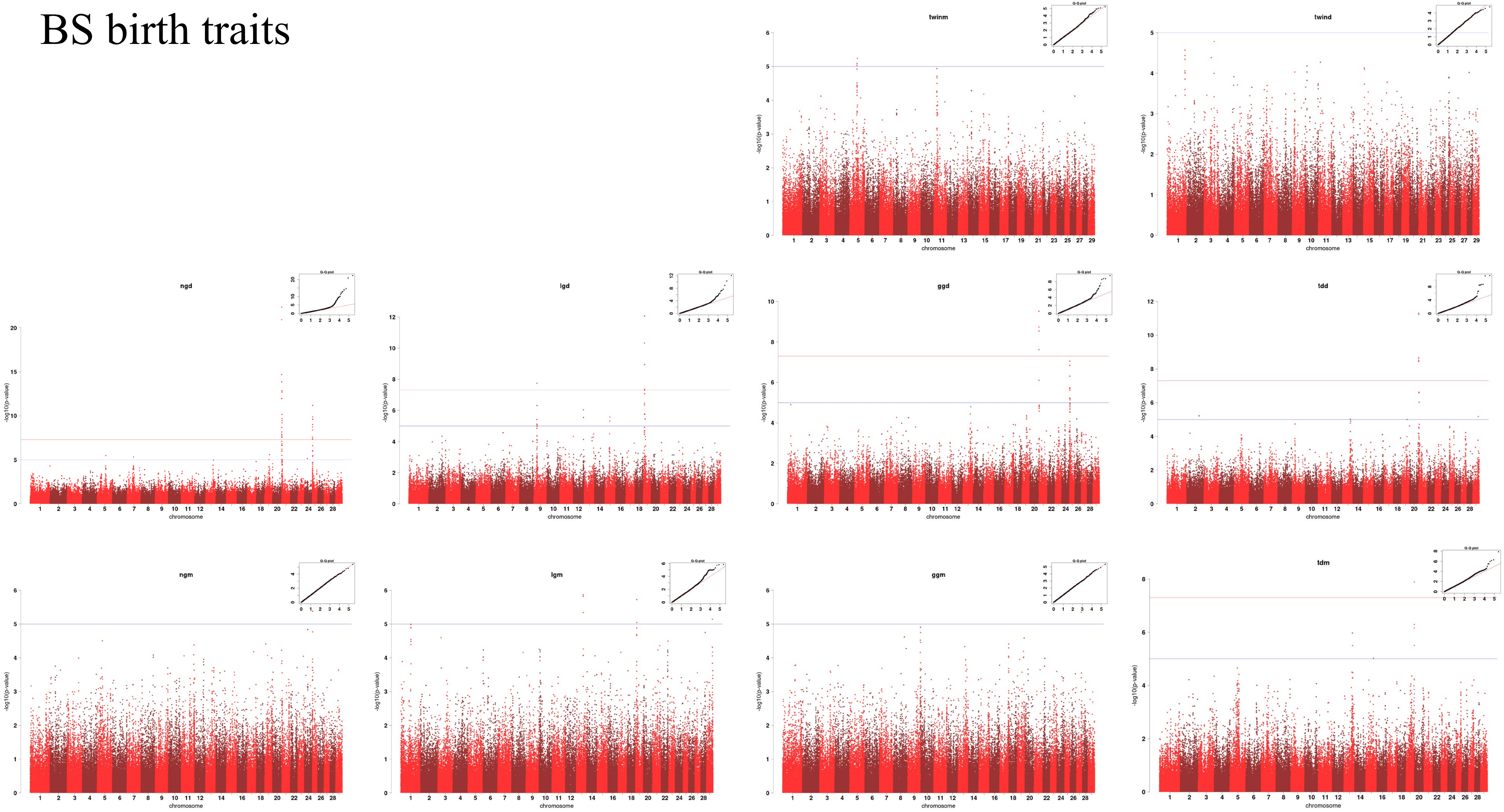

BS growth-related traits

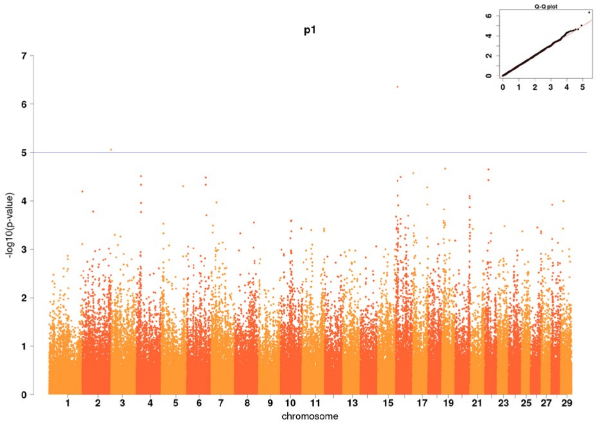

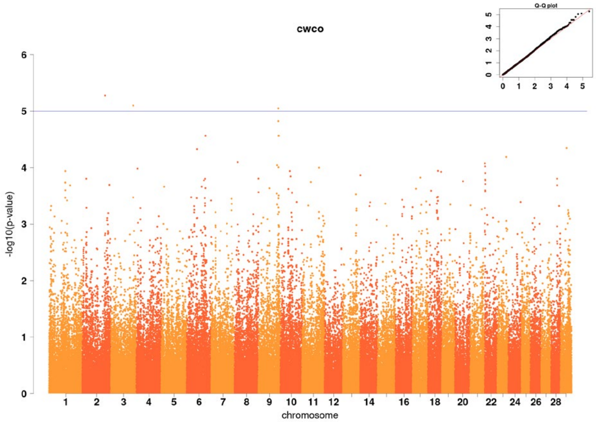

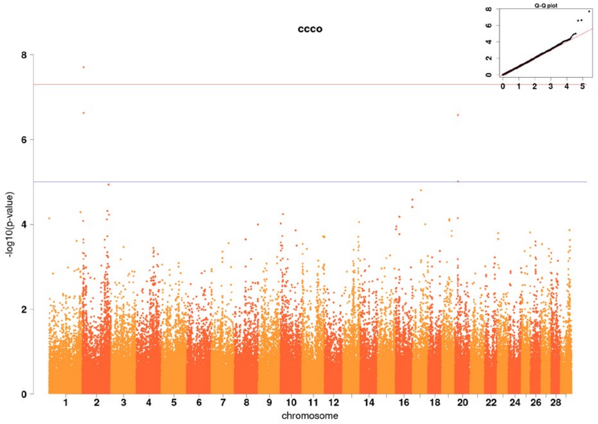

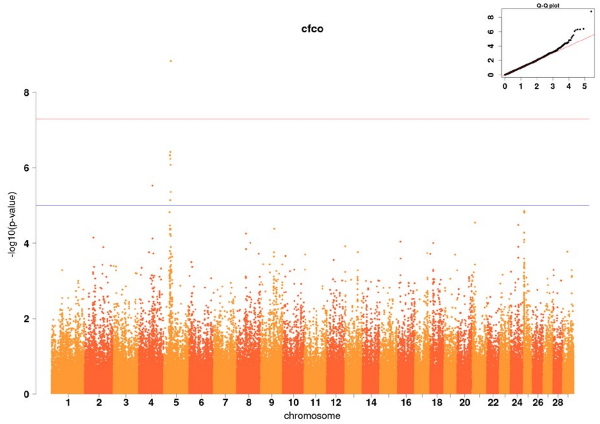

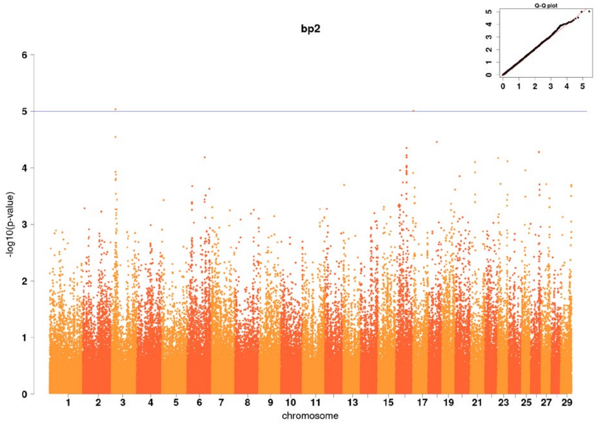

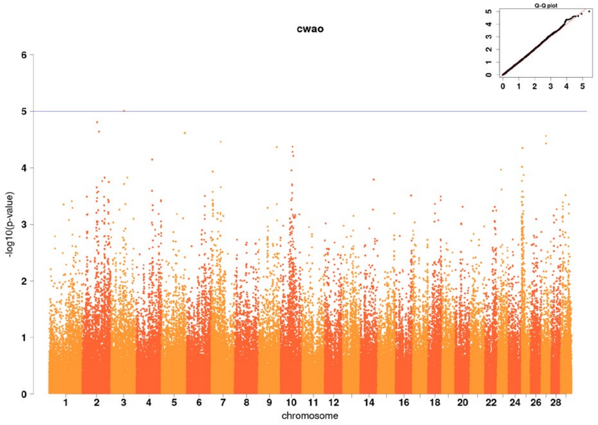

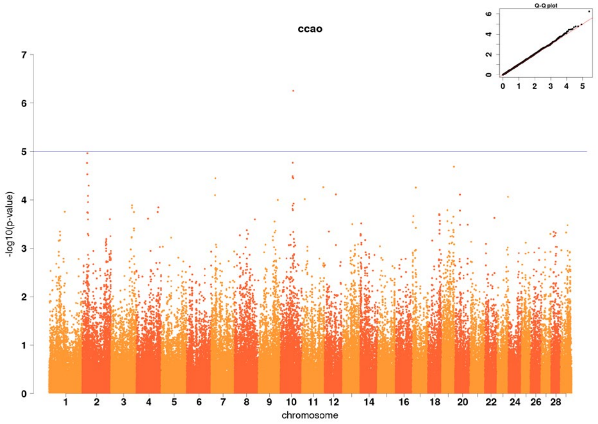

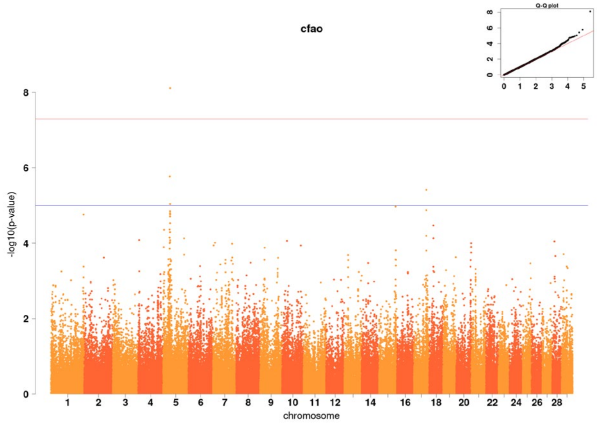

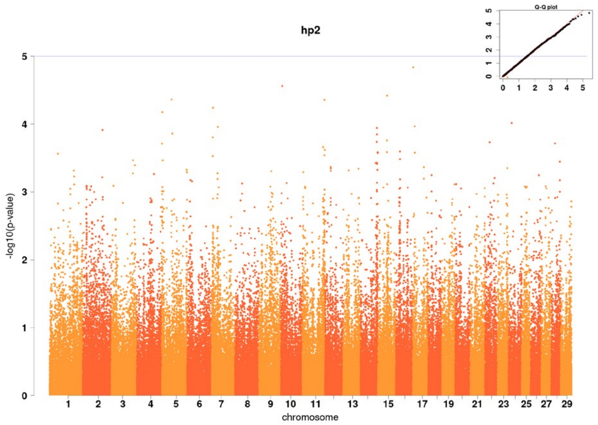

OB fertility traits

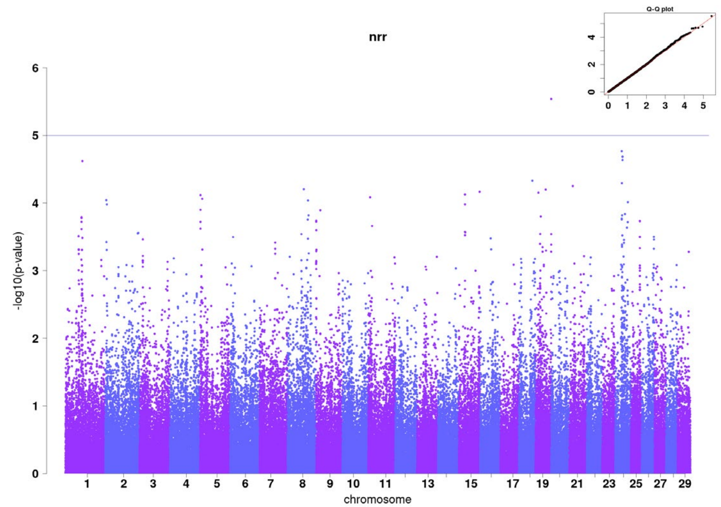

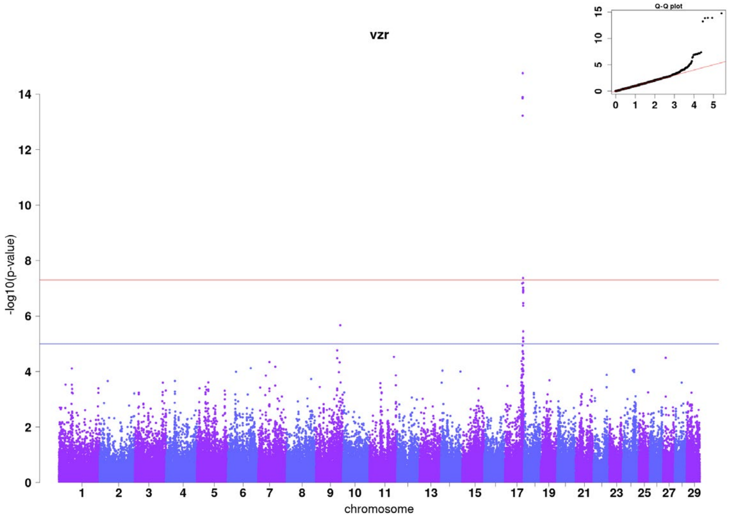

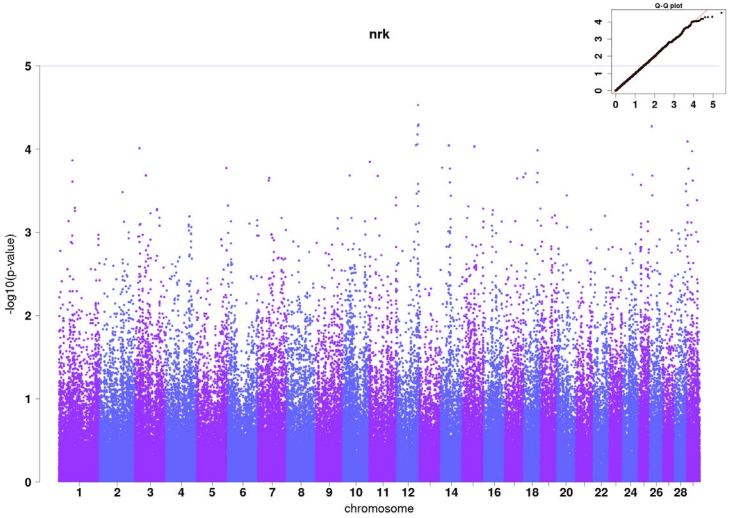

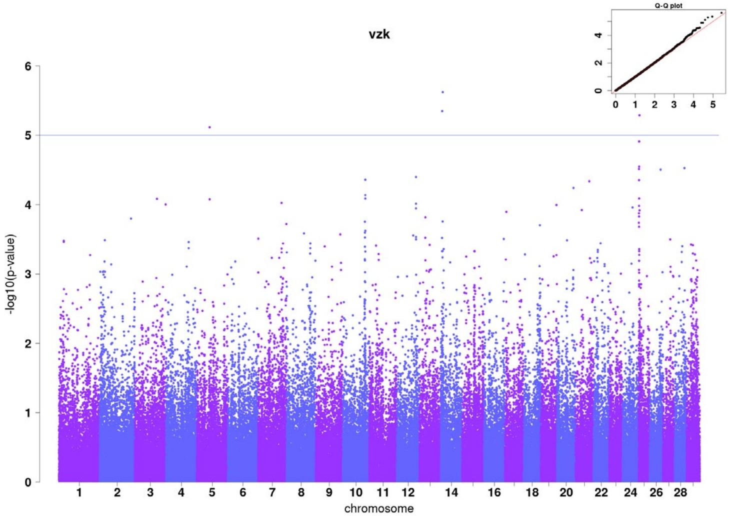

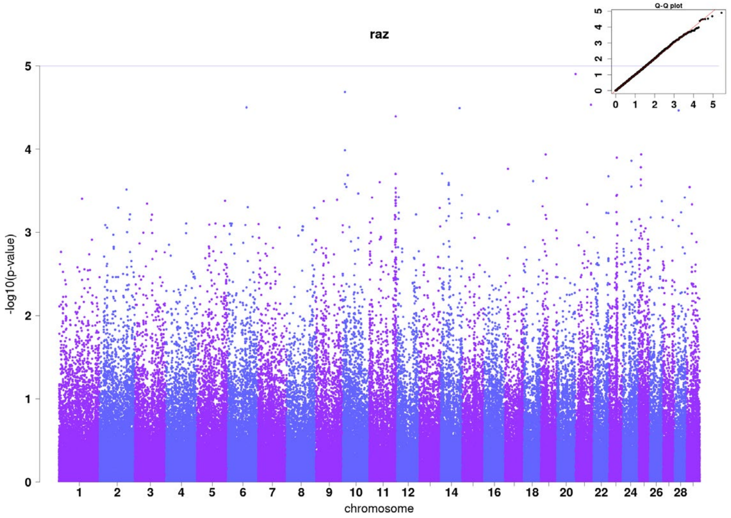

OB birth traits

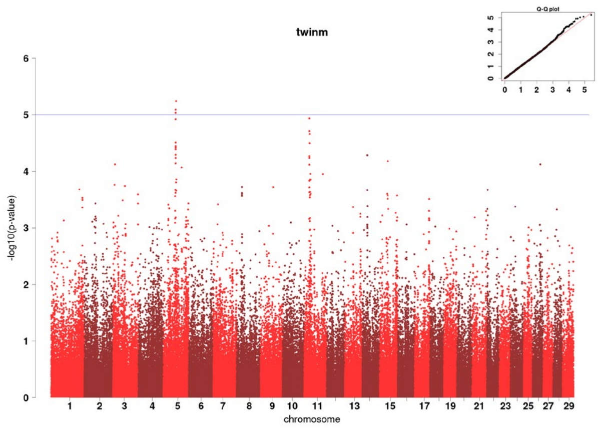

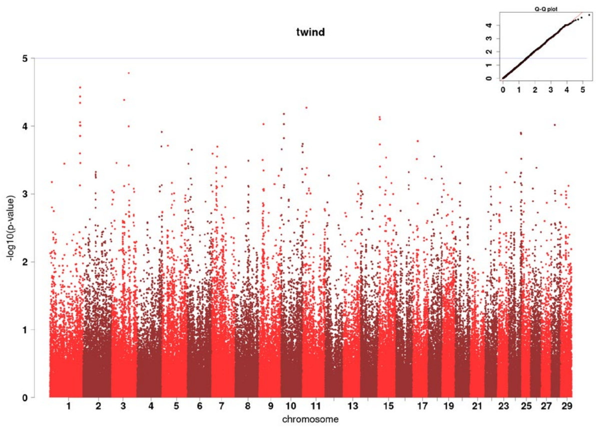

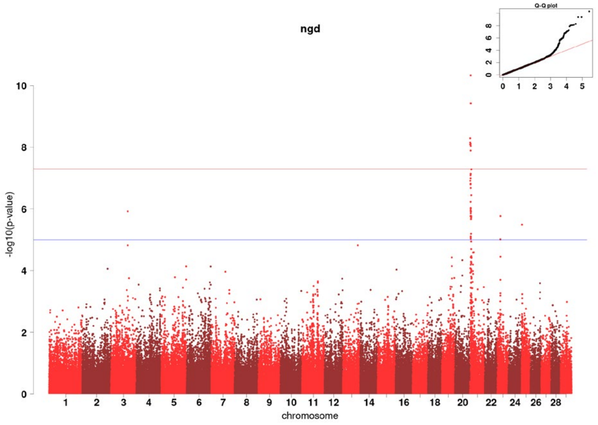

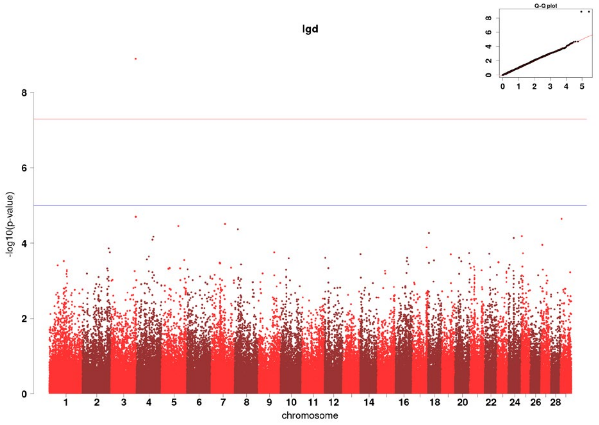

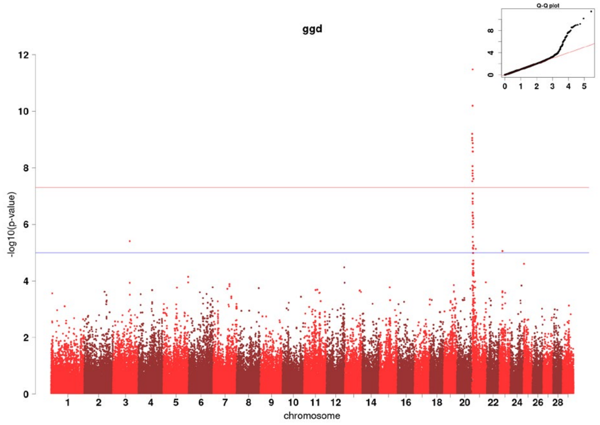

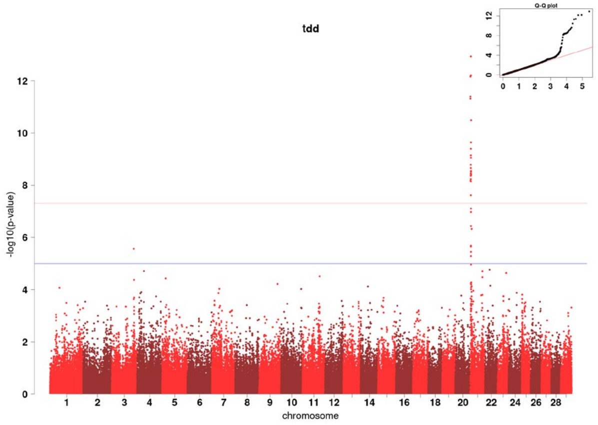

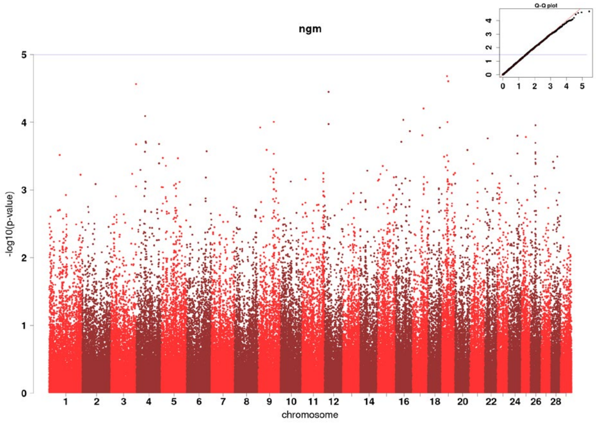

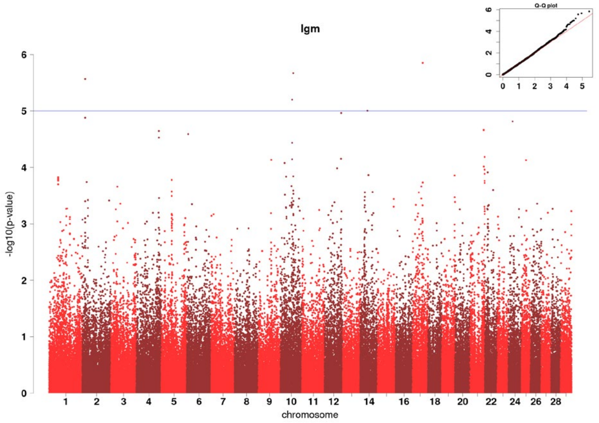

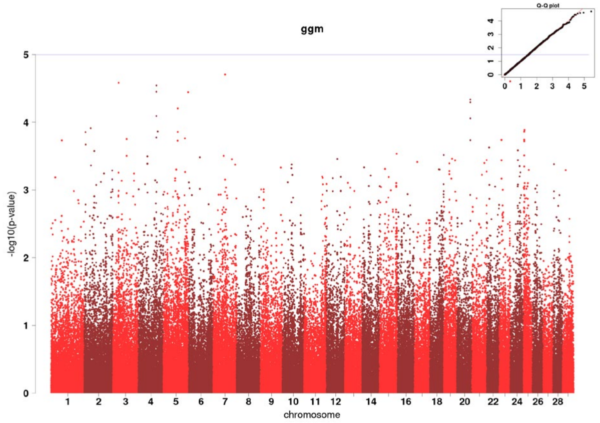

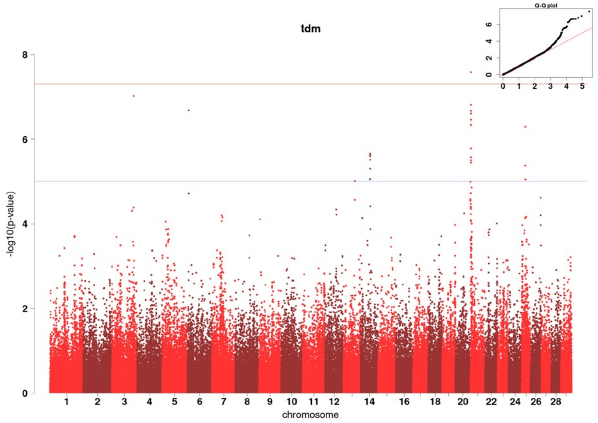

OB growth-related traits

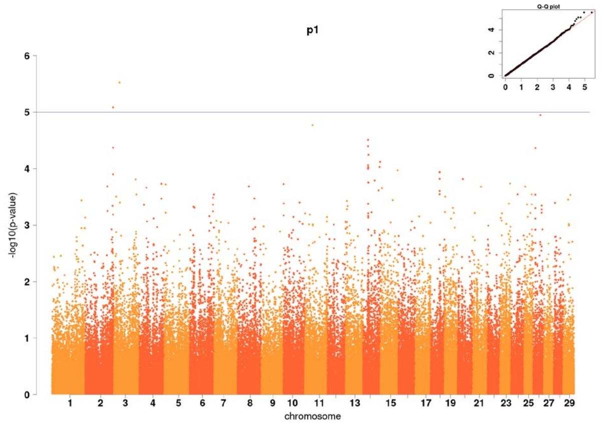

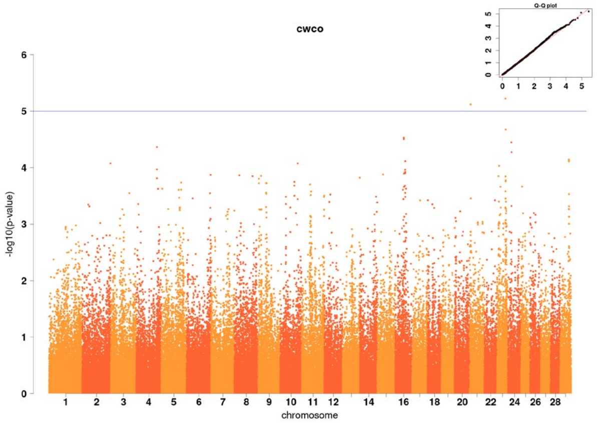

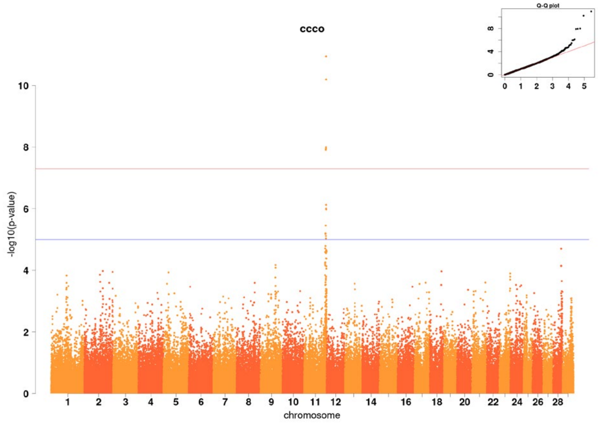

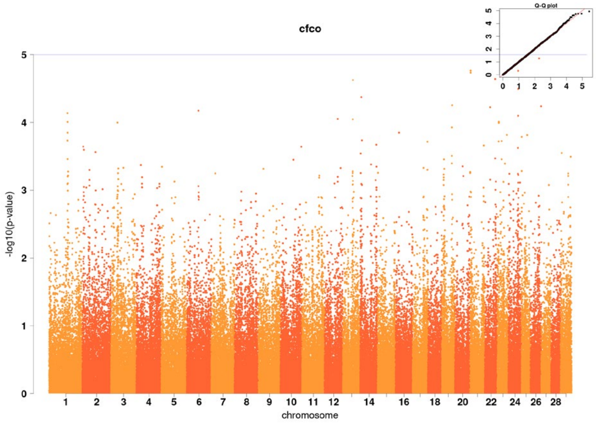

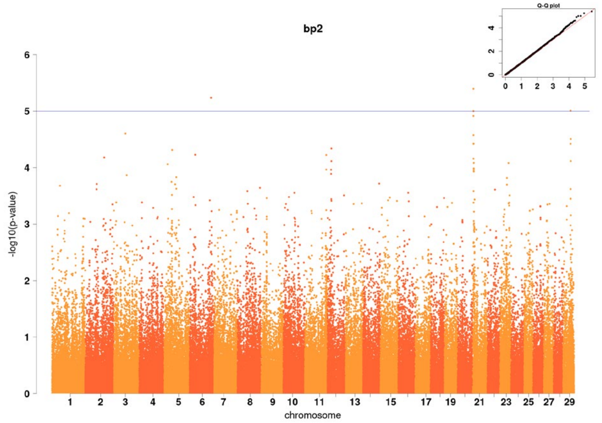

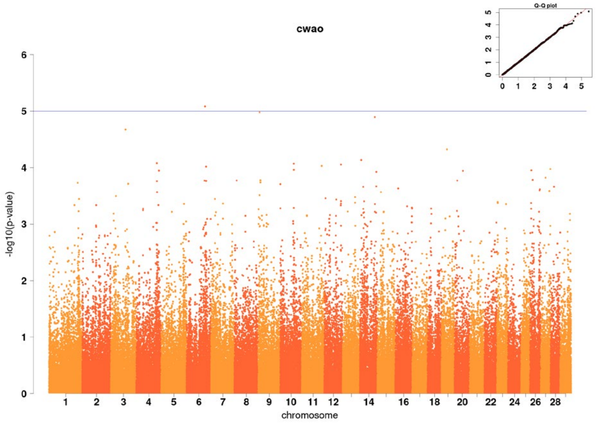

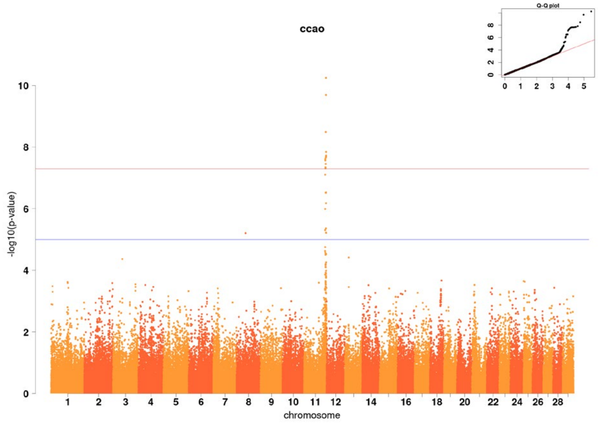

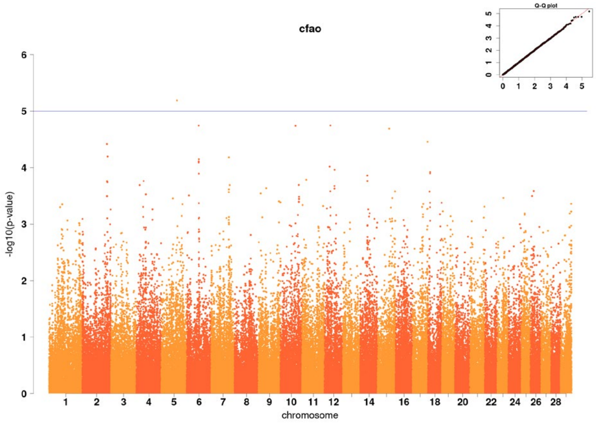

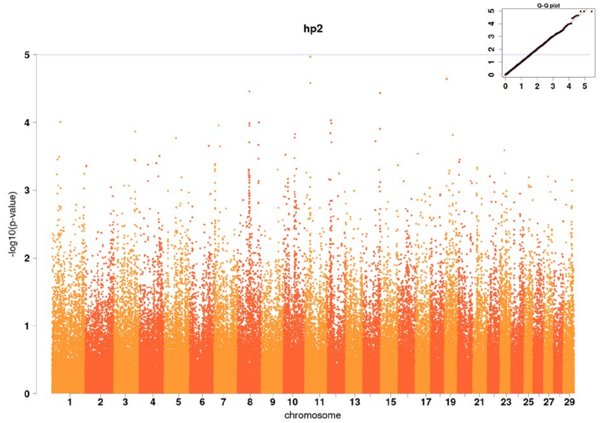

Supplement: Supplementary file 4 — Additional file 4: Figure S1. Manhattan plots and their QQ-plots of the GWAS results for the BS and OB populations. There is a page for each of the fertility, birth and growth-related trait groups, including a Manhattan plot for every single trait according to Table 2. [file 12711_2021_686_MOESM4_ESM.pdf]
